# Handgrip strength as an instrument for assessing the risk of malnutrition and inflammation in hemodialysis patients

**DOI:** 10.1590/2175-8239-JBN-2019-0177

**Published:** 2020-07-15

**Authors:** Caroline Finger Sostisso, Mayara Olikszechen, Melissa Nihi Sato, Miriam de Aguiar Souza Cruz Oliveira, Scheila Karam

**Affiliations:** 1 Fundação Pró-Renal, Setor de Nutrição, Curitiba, PR, Brasil.

**Keywords:** Muscle Strength Dynamometer, Muscle Strength, Nutrition Assessment, Malnutrition, Renal Dialysis, Dinamômetro de Força Muscular, Força Muscular, Avaliação Nutricional, Desnutrição, Diálise Renal

## Abstract

**Indroduction:**

Establishing which parameters to use for diagnosing malnutrition in hemodialysis patients is a challenge in clinical practice. The handgrip strength (HGS) has stood out as a method of assessing nutritional status. Thus, the aim of this study was to determine the cut-off point for HGS in the assessment of the risk of malnutrition and inflammation in HD patients, and its association with other parameters.

**Methods:**

Study carried out in hemodialysis units in the city of Curitiba, Brazil. We obtained the cut-off point of the HGS through the ROC curve, using the malnutrition and inflammation score (MIS) as a reference. We checked the relationship (Odds ratio) between the variables “MIS” and “HGS” with the other study variables using the multivariate analysis (logistic regression).

**Results:**

We assessed 238 patients (132 men), between 18 and 87 years of age (median = 59). The HGS cut-off point for diagnosing malnutrition and inflammation according to the reference used was <14.5 kg for women, and <23.5 kg for men. According to the HGS criteria, malnourished patients were older (OR = 0.958), with lower arm circumference (OR = 1.328) and higher scores in the malnutrition and inflammation score (OR = 0.85).

**Conclusion:**

HGS was significantly correlated with other nutritional assessment parameters. These results suggest that HGS is a valid screening tool to identify the risk of malnutrition and inflammation in hemodialysis patients.

## Resumo

**Introdução:**

Estabelecer quais parâmetros utilizar para diagnóstico de desnutrição em pacientes em hemodiálise (HD) é um desafio na prática clínica. A força de preensão manual (FPM) tem-se destacado como um método de avaliação do estado nutricional. Assim, o objetivo deste estudo foi determinar o ponto de corte da FPM na avaliação do risco de desnutrição e inflamação de pacientes em HD e sua associação com outros parâmetros.

**Métodos:**

Estudo realizado em unidades de hemodiálise na cidade de Curitiba, Brasil. Ponto de corte da força de preensão manual obtido através da curva ROC, usando como referência o escore de desnutrição e inflamação (MIS). A relação (Odds ratio) entre as variáveis “MIS” e “FPM” com as demais variáveis do estudo foi verificada por meio de análise multivariada (regressão logística).

**Resultados:**

Foram avaliados 238 pacientes (132 homens), entre 18 e 87 anos (mediana = 59). Ponto de corte da FPM para diagnóstico de desnutrição e inflamação de acordo com a referência utilizada: < 14,5 kg para mulheres e < 23,5 kg para homens. Pacientes desnutridos conforme critérios da FPM eram mais velhos (OR=0,958), com menor circunferência do braço (OR=1,328) e maior pontuação no MIS (OR=0,85).

**Conclusão:**

A FPM foi correlacionada de forma significativa com outros parâmetros de avaliação nutricional. Esses resultados sugerem que a FPM é um instrumento de triagem válido para identificar risco de desnutrição e inflamação em pacientes em hemodiálise.

## Introduction

Protein-energy malnutrition (PEW) is common in patients with chronic kidney disease (CKD), especially in those undergoing chronic hemodialysis (HD), being an important predictor of morbidity and mortality in this population.^(^ 1 ^-^ 3 ^)^

Although inadequate food intake contributes to this condition, there are other characteristics of the syndrome that cannot be explained only by anorexia, such as the inflammation affecting this population.^(^ 1 ^,^ 4 ^,^ 5 ^)^

The malnutrition-inflammation score (MIS) was developed from the Global Subjective Assessment (SGA), using the close relationship between malnutrition and inflammation among dialysis patients. In addition, higher MIS scores are associated with a higher risk of death and hospitalizations in HD patients.^(^ 6 ^-^ 8 ^)^

Despite these advantages, one of the MIS limitations is the low practicality for monitoring dialysis patients, since it uses subjective parameters, which require the evaluator’s experience, and depends on data that may not be available at a certain time.^(^ 3 ^)^

Handgrip strength (HGS) has stood out as a method of assessing nutritional status, and it is feasible in clinical practice.^(^ 9 ^)^ Still, since it is a test for measuring voluntary muscle strength, it is strongly correlated to body mass, making it possible to identify patients who had a significant reduction in nutritional status before any change occurred.^(^ 3 ^)^ In the scientific literature, few studies have evaluated HGS as a parameter for nutritional assessment in hemodialysis patients, and they used different measurement methods.^(^ 3 ^,^ 10 ^,^ 11 ^)^

In view of the aforementioned aspects, the objectives of the present study are to determine the cutoff point for HGS for men and women to identify the risk of malnutrition and inflammation, using MIS as a reference standard, and to evaluate its association with other nutritional parameters.

## Material and Methods

### Participants

This is a cross-sectional study carried out in four HD clinics (Pró-Renal Brasil) in the city of Curitiba, PR, Brazil. The eligible patients were over 18 years of age, of both sexes, had been on HD for at least three months and had no physical or cognitive limitations to perform the HGS measurement. The patients underwent three HD sessions per week, lasting three to four hours each, in the period between August 2016 and July 2017. We collected clinical, demographic and laboratory data from their electronic medical records (Dialsist®2017). The Human Research Ethics Committee of the Paranaense Institute of Otorhinolaryngology approved this study, and all participants signed an Informed Consent Form.

### Handgrip Strength (Hgs)

We measured HGS using a dynamometer, with a digital display with strength assessment measured by kilogram strength (maximum 90 kg). To use the test, the participant remained in an orthostatic position, with the elbows flexed at a 90-degree angle, with no place of support at the time of measurement. We ran the measurements after the second HD session of the week, on the upper limb contrary to the vascular/catheter access, making with three measurements and picking the maximum value of the three.^(^ 12 ^)^

### Malnutrition-Inflammation Score - Mis Score

We used the MIS according to the recommendations from Kalantar-Zadeh et al.,^(^ 6 ^)^ which use seven components of the original SGA^(^ 13 ^)^ adding the number of years of dialysis therapy, body mass index (BMI), serum albumin level and total iron binding capacity. We learned the serum albumin values ​​and total iron binding capacity from the electronic medical records (Dialsist®2017) for the month of the evaluation. We chose a severity level for each of the ten MIS components, which could range from 0 (normal) to 3 (severely abnormal). The sum of all these components could vary from 0 (normal) to 30 (severely malnourished).^(^ 6 ^)^

### Nutritional Assessment

A trained examiner took the anthropometric measurements. We calculated the BMI using the ratio between body weight (kg) and the squared height (m^2^), with the result expressed in kg/m^2^.^(^ 14 ^)^ To calculate the BMI, we considered the patient’s dry weight. To measure the arm circumference (AC), we used an inelastic measuring tape, considering the midpoint between the acromion and the olecranon, with the elbow flexed at 90º, in the arm opposite to the fistula or catheter. We ran the AC measurement before the hemodialysis session, since there is no consensus on the best time for such measurement.^(^ 15 ^,^ 16 ^)^ We analyzed the results and the adequacy of the AC according to Frisancho.^(^ 17 ^)^

### Statistical Analysis

Initially, we described the data considering the mean, standard deviation, minimum, maximum and quartile values for quantitative variables, and frequency tables for qualitative variables. We compared the groups of interest with the continuous variables, with normal distribution, using the Student’s t test, paired or unpaired, and the variables without normal distribution by the non-parametric Mann-Whitney test (or Wilcoxon test). We ran the association between qualitative variables using the chi-square test or Fisher’s exact test.

We ran the Spearman’s correlation to assess the relationship between MIS and other nutritional parameters. The correlation was weak when the values ranged from 0 to 0.29; moderate, from 0.3 to 0.69; and strong, from 0.7 to 1.0.

We checked the relationship (Odds ratio) between the MIS and FPM variables with the other study variables through a multivariate analysis (logistic regression). We dichotomized the MIS variable using the cutoff point of 5, already established by other authors;^(^ 18 ^,^ 19 ^)^ We obtained the cutoff point of the FPM variable through the ROC curve, using the MIS classes as a reference. The variables with a significance level of up to 20%, in univariate analyzes, entered the selection of variables for logistic regression purposes.

We assessed variable normality using the Shapiro-Wilk test. We assessed all the tests considering a significance level of 5%.

## Results

The study included 132 men (56%), aged between 18 and 87 years (median = 59 years) and time on HD ranging from six months to 17 years (median = 25 months); 113 patients (47%) were over 60 years of age.

The CKD causes were diabetes mellitus (35%), hypertension (28%), chronic glomerulonephritis (11%), polycystic kidney disease (2%), multiple myeloma (10%) and other causes (14%).

Table 1 shows the main characteristics of the patients, with the results stratified by sex. The mean and median values for height and HGS, respectively, were higher in men than in women (P <0.001). Regarding the adequacy of dialysis, men had worse adequacy of dialysis than women did (P <0.001); however, both within the reference values.

**Table 1:** Anthropometric and demographic characteristics of the participants ^a^

	Total (n = 238)	Men (n = 132)	Women (n = 106)	*p* for gender comparison
**Age, years ^b^**	59 (18;87)	61.5 (18;87)	55 (20;85)	< 0.001
18-39 years (%)	26 (10.9)	11 (8.3)	15 (14.1)	0.022
40-59 years (%)	99 (41.6)	48 (36.4)	51 (48.1)	
≥ 60 years (%)	113 (47.5)	73 (55.3)	40 (37.8)	
**Nutritional Parameters**				
Height, cm (mean ± SD)^c^	162.85 ± 10.09	168.16 ± 7.79	156.24 ± 8.63	< 0.001
BMI, kg/m^2^	24.94 (22.22;28.64)	24.84 (22.21;28.33)	25.15 (22.37;30.08)	0.38
AC, cm	28.5 (25.5;32)	28.5 (26;31)	28 (25;32)	0.896
% AC Adequacy	91.44 (83.61;102.52)	89.96 (81.25;98.29)	93.48 (84.4;108.90)	0.016
Albumin, g/dL	4.37 (4.07;4.64)	4.44 (4.10;6.64)	4.26 (4.01;4.61)	0.034
HGS, kg	20.5 (15.7;28.4)	26.6 (20.10;30.92)	15.9 (13;19.60)	< 0.001
**Renal function**				
Kt/V (mean ± SD)^c^	1.39 ± 0.43	1.31 ± 0.4	1.49 ± 0.46	0.001
Months on dialysis	25 (13;54)	24 (12.5;48)	25 (13;60)	0.356
Hemoglobin, g/dL	11.4 (10.2;12.5)	11.6 (10.35;12.8)	10.8 (9.8;12.4)	0.057

BMI, body mass index; AC, arm circumference; HGS, handgrip strength

a Median values (1st quartile; 3rd quartile) - Mann-Whitney test (significance level of 5%)

b Absolute values and relative frequency for categorical variables - Chi-square test (5% significance level)

c Values presented as mean ± SD - T-Student test (5% significance level)

Table 2 shows moderately negative correlations of MIS with HGS and albumin; and weak negative correlations of MIS with BMI, AC, significantly (p <0.05).

**Table 2:** Correlation between Malnutrition and Inflammation Score (MIS) with other variables

Variable	r	*p*
Age, years	0.107	0.111
BMI, kg/m^2^	-0.188	0.005
AC, cm	-0.261	<0.001
HGS, kg	-0.329	<0.001
Albumin	-0.393	<0.001
Months on dialysis	0.084	0.213

BMI, body mass index; AC, arm circumference; HGS, handgrip strength Spearman correlation (5% significance level)

We analyzed the ROC curve to establish the best cut-off point for HGS capable of identifying patients at higher risk of malnutrition-inflammation. The area under the ROC curve for HGS as a predictor of MIS was 73% (95% CI = 64% to 85%) for men, and 61% (95% CI = 49% to 74%) for women. The best cutoff point for HGS for men was 23.5 kg (sensitivity = 70%; specificity = 70%) and 14.5 kg for women (sensitivity = 70%; specificity = 50%).

The estimated prevalence of malnutrition based on these cutoff points was 36% in women and 39% in men. By MIS, malnutrition was 28% for women and 21% for men.

Table 3 depicts the association between the age, AC, MIS and HGS, variables with nutritional status, according to the MIS and HGS classification.

**Table 3:** Association between Malnutrition and Inflammation Score (MIS) and Handgrip Strength (HGS) by logistic regression in men and women, according to malnutrition criteria^a^

	MIS			FPM	
	Men	Women		Men	Women
Age (years)			Age (years)		
Nourished (≤5)	59.04±13.63	55.18±13.43^b^	Nourished (F >=14.5 e M >= 23.5)	57.38±14.14	53.41±13.51^b^
Malnourished (>5)	66.32±11.08	53.87±13.02^b^	Malnourished (F <14.5 e M < 23.5)	65.67±10.46	57.53±12.53^b^
*P*=	0.01	0.644	*P*=	0.01	0.123
Mid-arm circumference (MAC - cm)			Mid-arm circumference (MAC - cm)		
Nourished (<=5)	29 (26;31.25)	28 (25.25;33.75)	Nourished (F >=14.5 e M >= 23.5)	29 (27;31.5)	27.5 (25;34)
Malnourished (>5)	27 (22.75;29.25)	27.75 (23.5;31.5)	Malnourished (F <14.5 e M < 23.5)	27 (23;30)	29 (24;31.25)
*P*=	0.005	0.159	*P*=	0.004	0.696
Handgrip strength (FPM - Kg)			MIS		
Nourished (<=5)	27.86±8.75^b^	16.9 (13.6;20.05)	Nourished (F >=14.5 e M >= 23.5)	3 (1;4)	4 (2;5)
Malnourished (>5)	21.24±5.82^b^	14.15 (11.5;16.7)	Malnourished (F <14.5 e M < 23.5)	4 (3;7.5)	4 (3;7)
*P*=	<0.001	0.071	*P*=	<0.001	0.034

a Median values (minimum, maximum) - Mann-Whitney test (significance level of 5%)

b Values presented as mean ± OD - T-student test (5% significance level)

The men classified as malnourished by MIS were significantly older, with lower AC and less strength (p = <0.05). The same occurred with men classified with malnutrition by the HGS, they were significantly older and with lower AC, (p <0.05). Both malnourished men and women according to HGS had higher MIS scores. Among women, the other variables did not behave in this way and did not show statistical significance.

Tables 4 and 5 show the results of the linear regression between MIS and HGS, respectively, with the variables that were significant after comparison using the statistical model.

**Table 4:** Factors associated with MIS malnutrition, according to odds ratio (OR) values, 95% confidence interval (CI)

Nutritional Parameters	Total			Men (MIS>5)			Women (MIS>5)	
	Odds Ratio (95% CI)	*p*		Odds Ratio (95% CI)	*p*		Odds Ratio (95% CI)	*p*
Weight	0.968 (0.945-0.991)	0.008		-			-	
Urea	0.988 (0.978-0.997)	0.014		0.974 (0.959-0.990)	<0.001		-	
HGS*	0.295 (0.156-0.559)	<0.001		0.175 (0.064-0.477)	<0.001		0.382 (0.159-0.916)	0.031

HGS, handgrip strength

* HGS: ≥14.5 women; ≥ 23.5 men.

**Table 5:** Factors associated with HGS malnutrition, according to odds ratio (OR) values, 95% confidence interval (CI)

Nutritional Parameters	Total			Men (FPM<23.5)			Women (FPM<14.5)	
	Odds Ratio (95% CI)	*p*		Odds Ratio (95% CI)	*p* value		Odds Ratio (95% CI)	*p*
Age	0.958 (0.936-0.980)	<0.001		0.961 (0.929-0.995)	0.023		-	
AC	1.328 (1.033-1.707)	0.027		1.129 (1.012-1.259)	0.029		-	
MIS	0.85 (0.765-0.944)	0.002		0.823 (0.704-0.963)	0.015		0.382 (0.159-0.916)	0.031

AC, arm circumference; MIS, Malnutrition Inflammation Score

Table 4 shows that HGS ≥ 23.6 kg for men and ≥ 14.5 kg for women was significantly associated with a reduction in the odds ratio for malnutrition, especially in men. Increased weight and pre-dialysis serum urea were also associated with lower malnutrition OR.

Regarding the presence of malnutrition according to the HGS criteria (Table 5), advanced age and higher MIS scores were associated with malnutrition; the increase in AC was associated with a lower probability of malnutrition.

## Discussion

We found that men had higher HGS medians than women, which corroborates the results of other studies, both in healthy individuals^(^ 12 ^,^ 20 ^)^ and also in HD individuals.^(^ 3 ^,^ 8 ^,^ 10 ^,^ 11 ^,^ 21 ^)^ The difference in HGS between men and women is mainly associated with differences in body composition, with men generally having a greater amount of muscle mass, which directly reflects greater muscle strength.^(^ 11 ^,^ 22 ^)^

This HGS variation was evident when analyzing the results of the ROC curve that indicated different cutoff points for each gender. The HGS value with the best compensation between sensitivity and specificity was 23.5 kg in men and 14.5 kg in women. Garcia et al. suggested higher cutoff points (<18 kg for women and <28.5 kg for men), using the same methodology as this study. The value of 23.4 kg proposed for women by Silva et al.^(^ 3 ^)^ was similar to that established for men in the present study, and higher than that found for women. Other studies established lower HGS cutoff points than those found in this study, for each gender, but used different reference standards other than MIS.^(^ 8 ^,^ 23 ^)^ When comparing the results of the present study with those of other authors, it is evident that, in addition to the difference in methods, considering the characteristic of the method used as a reference standard is of paramount importance, since MIS has a subjective character, which may interfere with the evaluator’s experience and does not correlate directly with the individual’s body mass.

Therefore, we must use and compare the cutoff points proposed for the diagnosis of malnutrition in the population of adult and elderly patients who undergo HD three times a week, and consider the methodology and the characteristic of the reference standard used, since there is no gold standard for nutritional assessment in HD patients. In addition, we still need studies capable of proposing HGS cutoff points according to categories other than gender.

In this study, the estimated prevalence of malnutrition was higher by the HGS criteria than by the MIS, for both genders. In addition, we found that HGS inversely correlated with MIS and albumin, as noted by other authors.^(^ 3 ^)^ These findings support the use of HGS as a simple nutritional screening tool in patients undergoing HD. Still, studies indicate that malnutrition causes greater muscle fatigue, capable of altering muscle contraction and relaxation, contributing to lower HGS.^(^ 24 ^)^ Although MIS is a comprehensive nutritional screening tool and was developed specifically for renal patients, it depends on laboratory tests that are often unavailable at the time of evaluation.

CKD patients have higher levels of inflammatory biomarkers, such as C-reactive protein and interleukin 6, which have been associated with lower HGS and higher MIS. Concerning albumin, its usefulness to detect early changes in nutritional status cannot yet be confirmed. Although albumin is considered in MIS and is correlated with HGS, its low values ​​have been discussed in the population of renal patients on dialysis, since it may be more associated with inflammation than nutritional status.^(^ 4 ^,^ 25 ^-^ 28 ^)^ Thus, dynamometry is a more practical nutritional screening tool that can be used in the HD patient population.

HGS was the main variable associated with malnutrition, especially among men (OR = 0.175; p = 0.001). Using the logistic regression model, Silva et al.^(^ 3 ^)^ also investigated the validity of HGS as an instrument for identifying malnutrition through correlation with MIS, and they reported that each lower HGS standard deviation was significantly associated with a two-fold increase in the probabilities of an MIS ≥ 6, among men (odds ratio = 2.25, p <0.001) and women (odds ratio = 2.53, p <0.001). In the present study, men with higher HGS had 5.7 times less chance of presenting malnutrition (MIS ≥ 5), which may be associated with the fact that men had greater muscle strength than women.

The increase in pre-dialysis serum urea was another variable associated with a lower chance of malnutrition (OR = 0.988; p = 0.014), corroborating reports from other studies that low serum levels of urea in dialysis patients may reflect intake deficient in protein, skeletal muscle mass and related to a higher risk of death.^(^ 29 ^-^ 31 ^)^

In men, age was one of the variables significantly associated with malnutrition, by both criteria evaluated; malnourished men had a mean age higher than 65 years. In addition, for each year of life, men had a higher likelihood of malnutrition by 5% (OR = 0.961; p = 0.023). This influence of age on the malnutrition outcome is associated with several factors, but especially those related to the reduction of lean mass and muscle strength resulting from the various metabolic, hormonal and nutritional disorders of CKD, contributing to the increasingly prevalent sarcopenia in the elderly population.^(^ 22 ^)^ In their study, Sampaio et al,^(^ 32 ^)^ reported that the increase in age reflected the increase in MIS. Qureshi et al.,^(^ 33 ^)^ reported that elderly patients were often more malnourished than younger ones, according to the ASG, in addition to having lower HGS, similar to this study. Other studies have also found negative correlations between HGS and age.^(^ 10 ^,^ 21 ^,^ 34 ^,^ 35 ^)^

Another variable associated with malnutrition was MIS, especially among women (OR = 0.382; p = 0.031); and the higher the MIS, the greater the odds ratio for the patient to present malnutrition according to the HGS criteria. Other authors found a significant association between HGS and MIS.^(^ 3 ^,^ 21 ^)^ Lin et al.,^(^ 36 ^)^ when assessing sarcopenia in HD patients, found that patients with high MIS (≥ 7) had a 6.9-fold increase in the risk of sarcopenia when compared to those with low MIS, not differentiating between genders.

The variables presented and which were associated with malnutrition can be obtained in the routine nutritional assessment, enabling a quick identification of the nutritional risk. HGS, as well as the suggested cutoff points, stands out as a practical and easy instrument to use.

### Limitations

One of the methodological limitations of this study is the lack of an acceptable reference standard for MIS. We used the MIS value > 5 as the cutoff point in this study; however, other authors propose different cutoff points, according to the population evaluated.

It was not possible to assess whether there was a difference in HGS between patients who used the dominant arm or not, because we decided to use the arm without the arteriovenous fistula (AVF), to prevent the patient from applying the maximum hand grip pressure on the arm with the FAV.

Considering that we held this study in HD units in a single geographic region of the country, caution should be exercised when generalizing the findings to populations in other regions. The present study did not evaluate the results proposed by differences in age range - adults and the elderly - we assessed only the differences between genders. Further studies should take into account the age group to propose cutoff points for MIS and HGS, since aging causes changes in body composition. In addition, it is of utmost importance that new studies also use methods that assess quantity and/or quality of muscle tissue, since it is directly related to HGS.

## Conclusion

The best cut-off point for HGS capable of identifying patients at risk of malnutrition and inflammation was 23.5 kg for men and 14.5 kg for women. HGS was associated with malnutrition, according to the MIS classification, significantly in both genders. The results suggest that HGS is a valid screening tool to identify risk of malnutrition and inflammation in HD patients.
